# DualKG-DC: A Drug-Centric Dual-Layer Knowledge Graph Framework for Drug Combination Prediction

**DOI:** 10.1007/s10916-026-02416-y

**Published:** 2026-05-28

**Authors:** Zhenxiang Gao, Scott W. Perkins, Satya Parameswaran, Rong Xu

**Affiliations:** 1 Center for Artificial Intelligence in Drug Discovery, School of Medicine, Case Western Reserve University, Cleveland, OH USA; 2 Cleveland Clinic Lerner College of Medicine of Case Western Reserve University, Cleveland, OH USA; 3 Department of Biology, College of Arts and Sciences, Case Western Reserve University, Cleveland, OH USA

**Keywords:** Drug Combination, Knowledge Graph, Computational Prediction Framework

## Abstract

Most existing approaches to drug combination discovery are disease-centered, aiming to identify drug pairs for specific diseases. Complementarily, a drug-centered strategy starts from known drug combinations and explores new therapeutic indications, facilitating translational applications by leveraging combinations with established safety profiles. Here, we introduce DualKG-DC, a drug centered computational framework that provides a complementary perspective by identifying potential disease indications for a given drug combination. The dual layer knowledge graph architecture, which is pretrained on a foundation biomedical knowledge graph and subsequently refined on a task specific drug combination subgraph, may reduce reliance on large, labeled datasets by leveraging existing knowledge on drug targets, biological pathways, and observed phenotypic effects. In systematic benchmarking against three state-of-the-art models, DualKG-DC outperformed all comparison models, achieving an average Hits@10 of 0.48, MRR of 0.30, AUROC of 0.99, and AUPRC of 0.31. Notably, in cold start scenarios, DualKG-DC outperformed baseline methods in predicting indications for unseen drug combinations, achieving superior results with an average Hits@10 score of 0.32, an MRR of 0.18, an AUROC of 0.98, and an AUPRC of 0.23. These results highlight DualKG-DC as an effective platform for systematically discovering therapeutic opportunities of drug combinations. By leveraging a dual-layer architecture, the model enables effective knowledge transfer, enhancing predictive performance and robustness, particularly for previously unseen drug combinations.

**Supplementary Information:**

The online version contains supplementary material available at 10.1007/s10916-026-02416-y.

## Introduction

Drug combinations, which leverage multiple compounds with distinct mechanisms of action, hold significant promise for treating complex diseases such as neurodegenerative disorders, cancers, infectious diseases, cardiovascular diseases, and diabetes [1]. By targeting multiple pathways, these therapies offer enhanced therapeutic efficacy and reduced toxicity compared to single-agent treatments [2]. Despite this promise, identifying drug combinations with high clinical efficacy remains a significant challenge.

Computational approaches including network-based and machine learning approaches, have recently been developed to predict drug combinations for given diseases [3, 4]. Cheng et al. proposed a network-based methodology to predict the associations between disease and drug-drug combinations by quantifying the interactions between drug targets and disease proteins within the protein interactome [5]. Ding et al. developed a tucker tensor factorization model to predict synergistic drug combinations by leveraging knowledge of existing drug combinations, disease comorbidities, and disease treatments [6]. Recently, deep learning approaches have been proposed for drug combination prediction [7–10]. ComboNet is a neural network architecture designed to predict chemical synergy against SARS-CoV-2 by integrating a drug-target interaction (DTI) network with a target-disease association network [11]. AttenSyn applies an attention-based graph neural network to extract high-level latent features from molecular graphs and cell lines to predict the synergy of anticancer drug combinations [12]. Knowledge graphs (KGs) have emerged in recent years as powerful tools in the biomedical domain [13, 14]. KGANSynergy built a knowledge graph that integrates four types of biological data and employed a neural network-based multi-head attention mechanism to learn drug and cell line embeddings for predicting drug combination synergy [15].

Most existing computational approaches to drug combination discovery adopt a disease-centered perspective, in which a combinatorial number of drug pairs are screened for a given input disease, cancer cell line, or predefined set of disease-related targets (“disease looking for drug pairs”). In contrast, a drug-centered strategy offers a complementary paradigm: rather than exhaustively evaluating millions of potential drug pairs for each disease, it begins with a focused set of known drug combinations and systematically identifies new disease indications or conditions for which these combinations may be effective (“drug pairs looking for disease”). This is relevant for repurposing known drug combinations, as it aligns with a market expansion model that seeks additional indications for existing combinations. Because these combinations have already been studied, their safety profiles, pharmacokinetics, and potential drug-drug interactions are often better characterized, which may reduce certain aspects of clinical uncertainty and facilitate translational applications by leveraging established pharmacological knowledge and prior clinical experience.

In this study, we propose DualKG-DC, a drug-centered computational framework for drug combination prediction. Unlike traditional disease-centric approaches that search for drug combinations to treat a specific disease, DualKG-DC reverses this paradigm by identifying potential disease indications for a given drug combination. To account for the limited availability of labeled data for drug combinations, DualKG-DC uses a two-layer architecture. The first layer is trained on a foundational biomedical knowledge graph to learn broad drug related biological patterns, including drug targets, pathways, and pharmacological properties. The second layer uses a smaller, task specific sub-graph to adapt these representations to drug combination settings, based on available combination level information. This design enables reuse of established biological representations for inference on drug combinations and may reduce reliance on large, labeled datasets while supporting inductive prediction for novel or previously unseen drug pairs.

## Materials and Methods

**Fig. 1:**
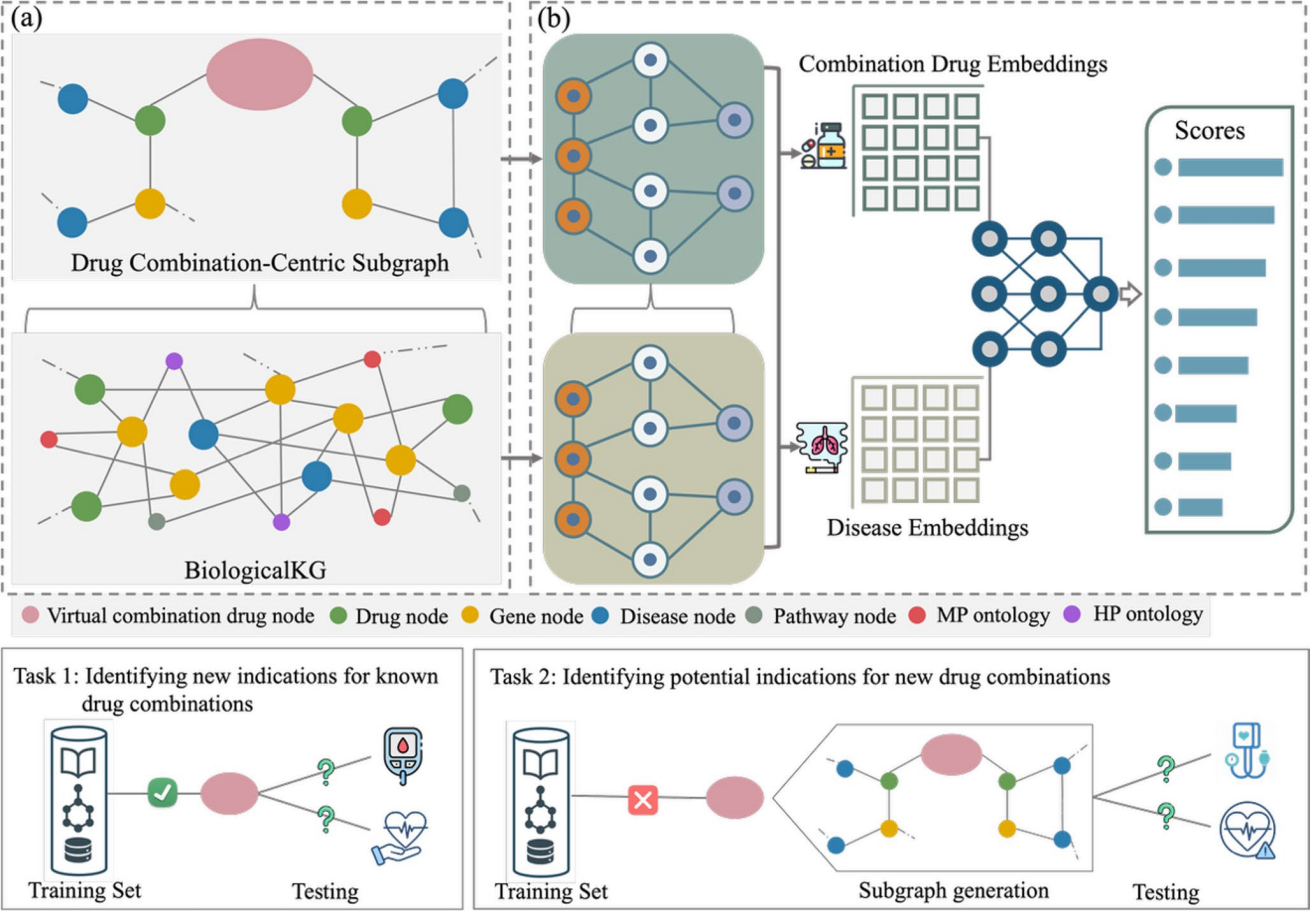
Overview of the DualKG-DC framework. (**a**) Two-layer Knowledge Graph Construction: The construction process began with the foundational biological knowledge graph (BiologicalKG), which integrates various biological knowledge bases to capture complex, multidimensional relationships among diseases, genes, drugs, pathways, and phenotypic annotations. From this, drug combination-centric subgraphs are then constructed by introducing a virtual combination node and extracting a two-hop neighborhood to capture combination-specific biological context. (**b**) The computational pipeline. The heterogeneous GNN encoder operated in two stages. First, it captured the heterogeneous topological structures and semantic features within the BiologicalKG to generate embeddings for its entities. These embeddings were then passed to the second layer, where message passing on the combination drug-centric subgraph generated the embeddings specifically for combination drug entities. Finally, the prediction module utilized these embeddings to compute association scores between combination drugs and diseases. Following the DualKG-DC framework, our study focuses on two prediction tasks: (1) transductive prediction, which aims to infer new disease indications for existing drug combinations that are present in the training set. In this setting, the model leverages learned representations of known combinations to predict previously unobserved indications; and (2) inductive prediction, which targets new and previously unseen drug combinations that do not appear during training. For each novel combination, a local subgraph is first constructed by incorporating the constituent drugs and their associated biological entities and relations. The trained model then applies learned message passing and aggregation functions to aggregate the knowledge information from the subgraph to generate an embedding for the new combination and infer its potential disease indications

In this section, we first briefly described knowledge graph construction and problem formulation, and then we introduced the detailed implementations of our framework (Fig. 1).

### Knowledge Graph Construction

DualKG-DC utilized a two-layer knowledge graph structure (Fig. 1): (i) a foundational biological KG named BiologicalKG that captured genotypic and phenotypic knowledge across diverse biological entities (ii) drug combination-centric subgraphs that focused specifically on biological knowledge involving drug combinations.

The BiologicalKG included associations between human genes and diseases [16], drug-target interactions [17], human protein-protein interaction networks [18], biological pathways relevant to human genes and diseases [19], phenotypic features associated with various conditions and human genes [20], and the relationships between human genes and mammalian phenotypes [21]. In total, the biological KG encompassed 45,709 entities interconnected by 935,694 interactions across eight distinct types of relations. Details of BiologicalKG was list in Table S1 in the supplementary file.

The second layer is built on top of the BiologicalKG and provides a drug combination-centric representation. For each queried drug combination, a task-specific subgraph is constructed by extracting a localized neighborhood from the BiologicalKG. A virtual combination node is first introduced to represent the drug pair as a unified entity and is connected to each constituent drug. The subgraph is then expanded to include nodes and relations within two hops from the virtual combination node. A two-hop expansion captures both direct drug-associated entities (e.g., targets and indications) and second-order biological context, including disease-associated genes and disease-related comorbidities. We selected a two-hop expansion because the foundation knowledge graph already encodes comprehensive biological relationships, allowing relevant knowledge to be transferred into the task-specific subgraph through message passing. A two-hop neighborhood allows connecting the drug combination node to both direct drug-associated entities and disease-relevant biological context. Disease comorbidities and known drug combinations with their indications are extracted with high precision from published biomedical research articles using previously developed iterative pattern-based natural language processing techniques [22, 23]. NLP-extracted knowledge is restricted to well-established biomedical relations, mapped to standardized biomedical identifiers, and derived from high-confidence sources. In our prior work [6], we demonstrated that the utility of NLP-extracted datasets of disease comorbidities and drug combinations can be effectively utilized for training and evaluating disease-centered drug combination prediction models. Detailed information is summarized in Table S2 of the Supplementary File.

### Problem Formulation

Given a heterogeneous knowledge graph $\:G=(V,E,R)$, we assume that node $\:i$ in the node set $\:V$, $\:i\:\in\:V$, edge $\:{e}_{i,j}=(i,r,j)$ in the edge set $\:E$, where $\:r\:\in\:\:R$ indicates the relationship type, and $\:i$ is called the head/source node and $\:j$ is the tail/target node. Each node in the knowledge graph is initialized with an embedding, denoted as $\:{h}_{i}^{\left(0\right)}$. For a given drug combination entity $\:i$ and a disease entity $\:j$, the primary objective is to predict the likelihood of the drug combination $\:i$ being indicated for the disease $\:j$ (see task 1 in Fig. 1). To enhance the model’s capability for inductive reasoning, we address scenarios involving novel drug combinations that are not present in the training set (see task 2 in Fig. 1). When a new drug combination is introduced, we constructed a subgraph that captures the surrounding knowledge graph context for this combination. By aggregating the knowledge information surrounding the new drug combination, our approach can inductively reason about and predict potential diseases that the new drug combination might treat.

### DualKG-DC Framework Overview

DualKG-DC is a deep learning framework composed of two complementary components that operate together (Fig. 1). First, a heterogeneous encoder learns meaningful representations (embeddings) for biological entities, including drugs, genes, diseases, and drug combinations, by modeling the complex relationships captured in the knowledge graphs. To accommodate the dual knowledge-graph structure, the encoder operates in two stages: it first learns general biological context from the BiologicalKG and then uses these representations to construct embeddings for drug combinations within drug combination-centric subgraphs. Second, a decoder module leverages the learned embeddings to infer potential therapeutic relationships. By jointly considering the embeddings of a drug combination, a disease, and their relation type, the decoder produces a score that reflects the likelihood that the combination is effective for the disease. This two-stage design mirrors the biological intuition that reasoning about drug combinations requires both broad biological knowledge, capturing how drugs, genes, and diseases interact, and combination-specific knowledge that reflects how individual drugs work together to produce joint effects.

#### Heterogeneous GNN Encoder

The purpose of the heterogeneous GNN encoder is to capture multi hop biological context, such as drug-target-pathway-disease relationships, within a unified embedding space. It learns node embeddings by iteratively applying nonlinear transformations that integrate relational context, thereby capturing biological knowledge encoded in neighboring relational structures. These transformations refine the embeddings by aggregating information from both neighboring nodes and their relations. In this work, we use CompGCN to learn foundational knowledge graph embeddings and extend it with an attention mechanism to generate embeddings for drug combinations [24–26].

##### Step 1

Initializing latent representations.

The initial node embedding for each node $\:i$, denoted as $\:{h}_{i}^{\left(0\right)}$, is initialized using Xavier normal [27] initialization. For the following message-passing process, the computation proceeds through two stages, referred to as steps 2–3.

##### Step 2

Node Embedding Generation in the BiologicalKG through Relationship-Specific Message Passing.

For capturing multi-hop dependencies in the BiologicalKG, the encoder stacks several convolutional layers, these layers compute the embeddings by aggregating the amount of information from neighboring entities and relations.

Message computation: let $\:{h}_{i}^{\left(l\right)}$ represents the input vector of the entity $\:i$ from the $\:(l-1)$-th layer. In the $\:l$-th layer, messages are computed from its neighbors $\:j$ based on the relationship type $\:r$:

$$\:{m}_{i,j}^{(l+1)}=\:\psi\:\left({h}_{j}^{\left(l\right)},{h}_{r}^{\left(l\right)}\right)\:$$

where $\:\psi\:\left(\bullet\:\right)$ is a composition operator, $\:{h}_{j}^{\left(l\right)}$ is the embedding of node $\:j$ from the previous layer, $\:{h}_{r}^{\left(l\right)}$ is the embedding of relation $\:r$ from the previous layer.

Message aggregation: the embedding of node $\:i$ in the $\:l$-th layer is updated by aggregating messages from $\:i$’s neighboring entities and relations:

$$\:{h}_{i}^{(l+1)}=f(\sum\:_{j\in\:N\left(i\right)}{W}_{\lambda\:}^{\left(l\right)}{m}_{i,j}^{(l+1)}\:+\:{W}_{o}^{\left(l\right)}{h}_{i}^{\left(l\right)})$$

where $\:f$ is an activation function, $\:{W}_{\lambda\:}$ is relation-specific coefficient matrix, $\:{W}_{o}$ is self-specific coefficient matrix. If there are a total of $\:l$ layers, the output $\:{h}_{i}^{l+1}$ is the final embedding of node $\:i$.

Relation embedding updating: let $\:{h}_{r}^{l+1}$ denote the representation of a relation $\:r$ after the $\:l$-th layer. Then,

$$\:{h}_{r}^{(l+1)}=\:{W}_{rel}^{\left(l\right)}{h}_{r}^{\left(l\right)}$$

where $\:{W}_{rel}$ is a learnable transformation matrix which projects all the relations to the same embedding space as entities.

##### Step 3

Combination Drug Embedding Generation via Message Passing in the drug combination centric subgraph.

For each drug combination, a subgraph is constructed which is centered on the combination node and including constituent drugs, their target genes, and associated diseases. The initial node embeddings in this subgraph are inherited from the BiologicalKG encoder. A multi-layer GNN with attention then propagates information within this subgraph. At each layer, the combination node aggregates messages from neighboring entities across different relation types. An attention mechanism assigns higher weights to biologically informative relations, enabling the model to focus on the most relevant signals when forming the combination embedding. This design allows DualKG-DC to generalize to novel drug combinations by learning how to aggregate biological evidence rather than memorizing specific combination patterns.

Starting with the embeddings derives from the BiologicalKG as the initial input, the message-passing function aggregates information from the neighboring entities of the combination drug entity in the subgraph. The aggregated message for node $\:v$ across all relation types $\:R\left(v\right)$ in the $\:l$-th layer is then:

$$\\\begin{array}{c}\:e_v^{(l+1)}=f\\\left(\sum\:_{u,r\in\:N\left(v\right)}{\alpha\:}_{u,r}m_{u,r,v}^{(l+1)}\right)\end{array}$$

where $\:N\:\left(v\right)$ is the set of immediate neighboring entities and relations of entity $\:v$, $\:f$ is a non-linear activation function. The attention weight $\:{\alpha\:}_{u,r}$ is computed using a softmax over relation-specific scores. The attention mechanism is implemented as a single-layer feedforward neural network parameterized by the weight matrix $\:{W}_{att}$, with a LeakyReLU non-linearity applied.

$$\\\begin{array}{c}\:{\alpha\:}_{u,r}=softmax\\\begin{pmatrix}\frac{exp\left(LeakyReLU\left(W_{att}\:m_{u,r,v}\right)\right)}{\sum\:_{i\in\:N\left(v\right)}\sum\:_{r^{'\:}\in\:R\left(v\right)}exp}\\\left(LeakyReLU\left(W_{att}\:m_{i,r^{'\:},v}\right)\right)\end{pmatrix}\end{array}$$

Then, the embedding of node $\:v$ in the $\:l$-th layer is updated by aggregating messages from $\:v$’s neighboring entities and relations:

$$\:{h}_{v}^{(l+1)}=f(\:{W}_{\lambda\:}^{\left(l\right)}{e}_{v}^{\left(l+1\right)}+\:{W}_{o}^{\left(l\right)}{h}_{v}^{\left(l\right)})$$

where $\:f$ is an activation function, $\:{W}_{\lambda\:}$ is relation-specific coefficient matrix, $\:{W}_{o}$ is self-specific coefficient matrix.

During training, we provide the model with a set of known drug combinations, each with its corresponding subgraph. For each subgraph, the model learns the optimal parameters by performing message passing and updating node embeddings. The loss function is computed on the final combination embeddings and backpropagated through the network. This process teaches the model how to aggregate information from the subgraph, rather than memorizing the specific structure of the training graphs. For inference on a novel drug combination, we construct its subgraph and initialize node features in the same way. The trained model then uses its learned aggregation and update functions to compute the final combination embedding.

#### Inferring Indications for Drug Combination

Predicting score calculation: the prediction module of DualKG-DC is designed to capture and leverage the biological information encoded in entity and relation embeddings to infer potential new disease indications for drug combinations, following the InteractE framework [28]. Formally, for a combination drug $\:i$, disease $\:j$ and relation $\:r$, the prediction score is calculated as follows,

$$\\\begin{array}{c}\:S_{i,r,j}\\=\left[Flatten\left(\sigma\:\left(BN\left(Conv\left(\Omega\:\left(h_i,h_r\right);\backslash\Theta\right)\right)\right)\right)\right]h_j\\\end{array}$$

where $\:\varOmega\:(\bullet\:)$ is a reshaping and feature-expansion function, $\:Conv(\bullet\:,\varTheta\:)$ is a convolution operation, $\:BN(\bullet\:)$ a batch normalization layer, $\:\sigma\:(\cdot\:)$ is a non-linear activation function, $\:Flatten(\cdot\:)$ is a reshaping step that converts the 2D feature map into a one-dimensional vector.

Training Objective: given the predicted probability $\:{\widehat{y}}_{i,j}$for a drug combination $\:i$ and disease $\:j$, and the corresponding ground-truth label $\:{y}_{i,j}\:\in\:\left\{\mathrm{0,1}\right\}$, the loss is defined as:

$$\:{\mathcal{L}}_{BCE}=\:-[{y}_{i,j}\mathrm{log}\left({\widehat{y}}_{i,j}\right)+\left(1-\:{y}_{i,j}\right)\mathrm{log}\left(1-\:{\widehat{y}}_{i,j}\right)]$$

where $$\:{\widehat{y}}_{i,j}=1/(1+\:{e}^{-{S}_{i,r,j}})$$.

Model parameters are optimized end-to-end using the Adam optimizer with L2 weight decay to mitigate overfitting.

### Implementation

DualKG-DC was implemented using PyTorch. The hyperparameter tuning was performed using the validation set through grid search, with optimal values selected based on filtered Mean Reciprocal Rank (MRR) results. The GCN layer depths ($\:l$) was set to 2. For embedding dimensions ($\:\kappa\:$), increasing $\:\kappa\:$ slightly improved performance but led to high memory and computation demands when overly large. Therefore, $\:\kappa\:$ was set to 200 as a balanced choice. The learning rate ($\:\eta\:$) and batch size ($\:\beta\:$) were set to 0.001 and 128, respectively. To prevent overfitting, dropout was applied after each convolution layer, with rates of 0.1 in the embedding module and 0.3 in the prediction module. This process was iteratively performed over 500 epochs. All models were trained using a single NVIDIA Tesla V100 GPU with 32 GB of memory.

### Evaluation and Comparison

#### Experimental Settings

We performed a 5-fold cross-validation to evaluate the model’s performance in predicting potential indications for combination drugs. To create negative samples for evaluation, we enumerated drug combination-disease associations that were not present in the ground truth and treated them as negative examples. Specifically, for each known triple $\:(i,r,j)$ in the test set, where $\:i$ represents a combination drug entity, $\:r$ is the relation “treats”, and $\:j$ is a disease entity, we generated a set of negative samples $\:(i,r,v)$. These were created by replacing the disease entity $\:j$ with another disease entity $\:v$, where $\:(i,r,v)$ was not part of the ground truth. To construct a comprehensive negative dataset, we enumerated all possible disease entities for each test triple.

For evaluation, we conducted experiments under the following scenario:

- Transductive reasoning: all combination drug entities and disease entities present in the test set were also observed during training and the model inferred interactions among known entities. To evaluate the model’s ability to predict potential indications for known drug combinations, we shuffled all associations between combination drugs and diseases and divided the data into 60% training, 20% validation, and 20% testing subsets. This process was repeated to create five distinct splits for cross-validation experiments. The model was trained on the 60% training subset to learn patterns among entities, fine-tuned on the 20% validation subset, and evaluated on the 20% test subset to assess its performance.
- Inductive reasoning: the test set includes combination drug entities that were unseen during training. This setup assessed the model’s generalization ability to handle new, previously unseen entities by leveraging the learned knowledge and context from the training data. In this setting, 5-fold cross-validation was conducted to ensure robustness. The training set included only 80% partition of known combination drug entities, while fine-tuning was conducted on a separate validation subset drawn from these known entities. The model’s performance was then evaluated on a held-out test set consisting of 20% entirely unseen drug combination entities, with no overlap of constituent drugs between the training and test sets, to assess its ability to effectively handle inductive prediction scenarios.

To evaluate the performance of the model, we employed several widely recognized metrics, including Hits@N, Mean Reciprocal Rank (MRR), area under the receiver operating characteristic curve (AUROC), and area under the precision recall curve (AUPR). Filtered evaluation metrics were used in our study, in which all other known true triples for the same query are removed from the candidate set before ranking, thereby reducing bias caused by knowledge graph incompleteness. Hits@N measures the percentage of true triples in the test set that are ranked by the model within the top N positions, while MRR calculates the average inverse rank of true triples. AUROC assesses the model’s capability to distinguish between positive and negative samples across all classification thresholds, providing insight into the model’s overall discriminative power. AUPR evaluates the precision recall tradeoff, particularly in highly imbalanced settings.

#### Baselines

Current research in drug combination prediction predominantly focuses on drug synergy score prediction or drug combinations tailored for specific cell lines [2–4]. These approaches typically model how pairs of drugs interact in a disease- or cell-line specific context, with the goal of identifying combinations that exhibit synergistic therapeutic effects. While these methods have advanced the field, they are primarily disease-centric or cell-line-centric and rarely consider drug-centric representations that generalize across diseases or treatment contexts. To evaluate the performance of DualKG-DC, we selected three types of baseline methods for comparison.

TuSDC is a Tucker tensor factorization-based model for disease-centered drug combination prediction [6]. The model represents drug-drug-disease interactions in a tensor formulation and learns latent representations through factorization [29, 30]. It has been benchmarked against existing combination prediction methods and demonstrated competitive performance. For our evaluation, we re-implemented this approach, denoted as TuckerTD, and evaluated it on the same datasets to ensure a fair comparison.

KGANSynergy is a knowledge graph-based model for drug synergy prediction that adopts a graph attention network architecture [15]. Given a drug pair and a cell line, the model performs hierarchical propagation over a heterogeneous knowledge graph to collect multi-hop neighborhood information. At each propagation step, a multi-head attention mechanism is applied to weight neighboring nodes and aggregate their features, producing embeddings for drugs and cell lines. These representations are then concatenated and passed to a multilayer perceptron to compute the synergy score. Based on this design, we re-implemented the model, denoted as KGANCom, as a baseline and evaluated it on the same datasets used in DualKG-DC to ensure a fair comparison.

Since our framework is built upon knowledge graphs, we also compared against RotatE, a state-of-the-art embedding method for knowledge graphs. RotatE models relations as rotations in complex vector space and has demonstrated strong performance on link prediction and reasoning tasks across benchmark datasets [31]. Including RotatE provides a baseline to specifically assess the benefits of our knowledge graph-based design relative to established embedding methods.

## Results

### Performance in Transudative Learning: Predicting Potential Indications for known Drug Combinations

**Fig. 2:**
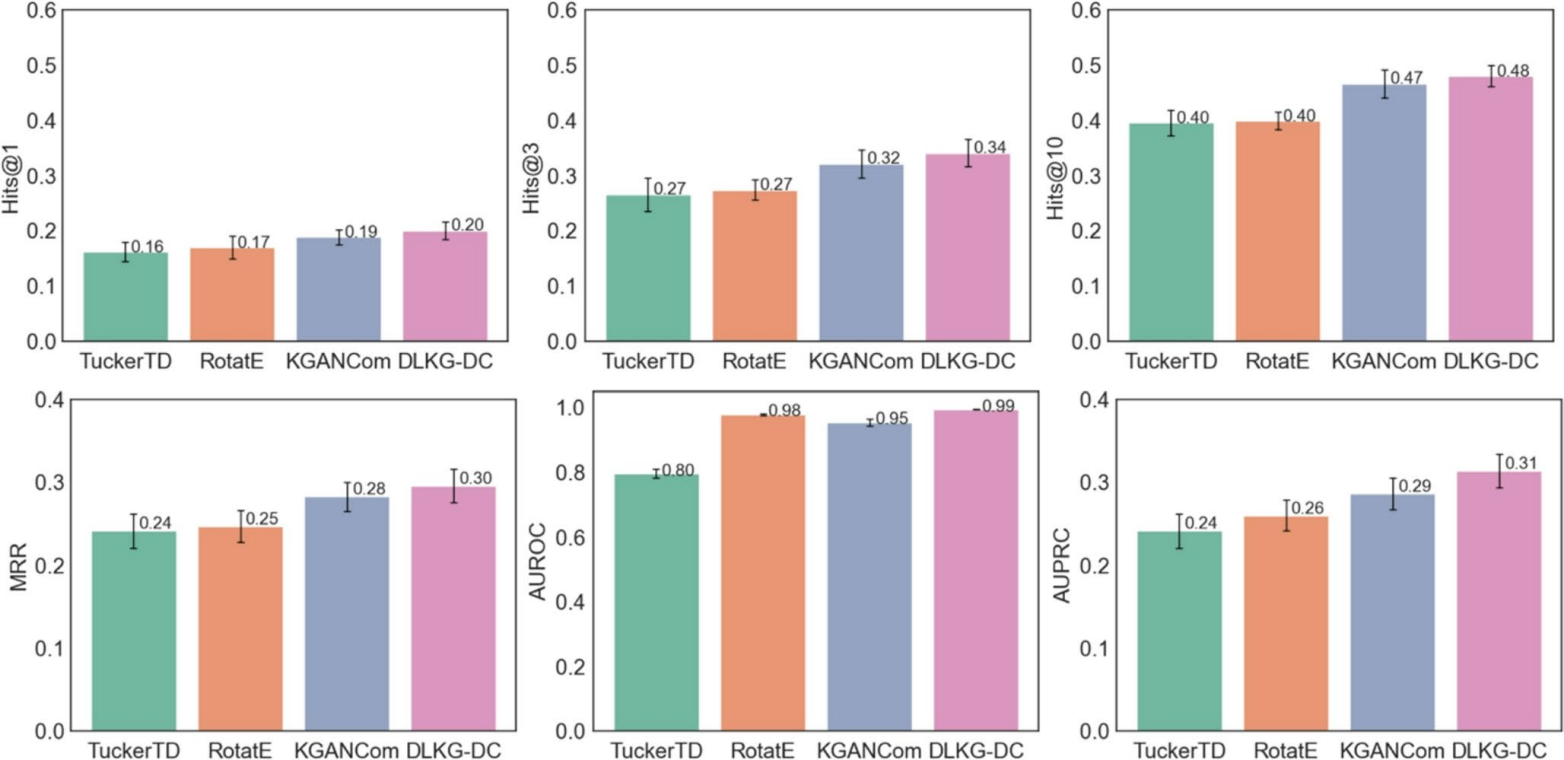
Performance evaluation of DualKG-DC for drug combination indication prediction under the transductive setting. Bars represent the mean performance across five independent runs, measured by Hits@1, Hits@3, Hits@10, MRR, AUROC, and AUPR. Error bars indicate the standard error (SE)

We first evaluated the performance of DualKG-DC against three baseline models using 5-fold cross-validation (Fig. 2). Among the baseline models, KGANCom model had an excellent performance with an average Hits@10 of 0.47, MRR of 0.28, and AUPRC of 0.29. RotatE, achieved the highest AUROC among the baselines, with an average score of 0.98. In comparison, DualKG-DC surpassed all baseline models across these evaluation metrics, achieving an average Hits@10 of 0.48, MRR of 0.30, AUROC of 0.99, and AUPRC of 0.31.

### Performance in Inductive Learning: Predicting Potential Indications for Novel Drug Combinations

**Fig. 3:**
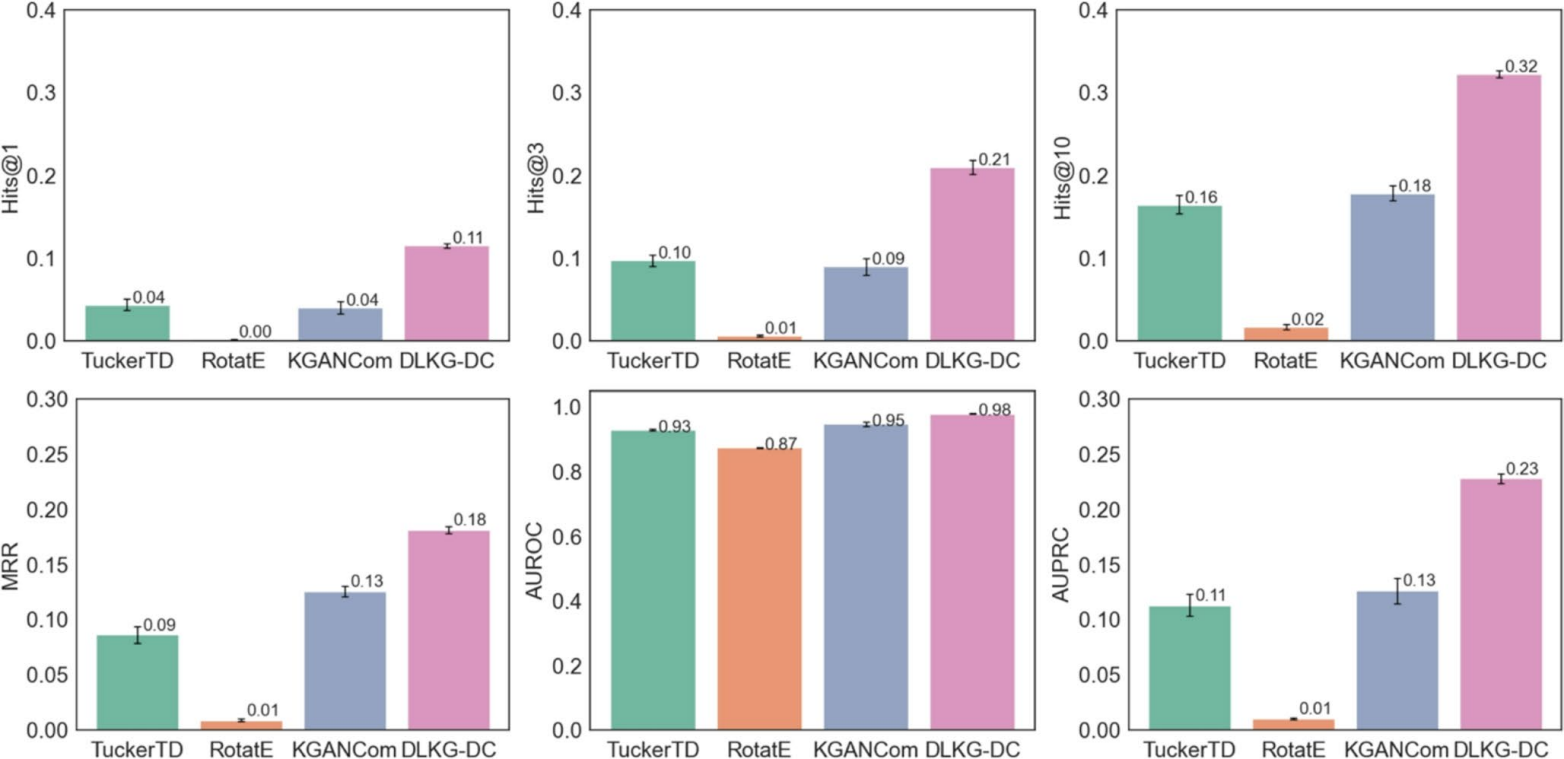
Performance evaluation of DualKG-DC for drug combination indication prediction under the inductive setting. Bars represent the mean performance across five independent runs, measured by Hits@1, Hits@3, Hits@10, MRR, AUROC, and AUPRC. Error bars indicate the standard error (SE)

To assess the ability of DualKG-DC to predict potential indications for new drug combinations, we compared its performance against several baseline methods. In Fig. 3, the results demonstrated that DualKG-DC achieved superior performance across all metrics, achieving superior results with an average Hits@10 score of 0.32, an MRR of 0.18, an AUROC of 0.98, and an AUPRC of 0.23. Compared to TuckerTD, the best baseline model, the AUROC of DualKG-DC was 5% higher, and the average scores of Hits@10, MRR, and AUPRC were twice than TuckerTD model.

### Ablation Study

DualKG-DC adopts a dual-layer knowledge graph architecture to enhance model generalization. To evaluate the contribution of this design, we conducted ablation experiments. Specifically, we developed two variants of DualKG-DC. The first variant, DualKG-DC-Onelayer, is a single-layer model trained on a unified knowledge graph that integrates all information from the BiologicalKG and the combination subgraphs into a single graph. The second variant, DualKG-DC-Subgraph, is a single-layer model trained solely on the drug combination-centric subgraph. These variants were compared with the DualKG-DC model on two tasks to assess the impact of the dual-layer architecture on generalization performance.

Table 1 summarizes the results of the ablation study. The DualKG-DC model, which employs a dual-layer architecture integrating both the BiologicalKG and the drug combination-centric subgraph, consistently outperforms its single-layer variants across both transductive and inductive settings. Compared with DualKG-DC-Onelayer, which aggregates all information into a single graph, DualKG-DC achieves moderate but consistent improvements across Hits@10, MRR, and AUPRC. These gains are more pronounced under the inductive setting, where DualKG-DC improves Hits@10 (0.32 vs. 0.29), MRR (0.18 vs. 0.16), and AUPRC (0.23 vs. 0.19), indicating better generalization to unseen combinations. In contrast, the DualKG-DC-Subgraph variant, trained only on the combination-centric subgraph, shows substantially lower performance, highlighting the importance of incorporating broader biological context.

**Table 1:** Results of model ablation experiment

Scenarios	Method	Hits@10	MRR	AUROC	AUPRC
Transductive reasoning	DualKG-DC-Subgraph	0.42	0.24	0.98	0.27
	DualKG-DC-Onelayer	0.47	0.29	0.99	0.30
	DualKG-DC	**0.48**	**0.30**	**0.99**	**0.31**
Inductive reasoning	DualKG-DC-Subgraph	0.18	0.11	0.96	0.12
	DualKG-DC-Onelayer	0.29	0.16	0.98	0.19
	DualKG-DC	**0.32**	**0.18**	**0.98**	**0.23**

### Case Study

To further validate the efficacy of our model, we conducted a case study to identify potential indications for a drug combination with an established safety profile. We selected the combination of metformin and atorvastatin, which has been developed and extensively studied for the treatment of type 2 diabetes accompanied by dyslipidemia [32]. Importantly, no indication labels related to this combination were used during training. Furthermore, drug pairs involving metformin or atorvastatin were excluded from the training set, ensuring that the model had no direct or indirect exposure to this drug combination.

Table 2 presents the top 5 highest-scoring potential disease indications predicted for the metformin-atorvastatin combination. The results highlight several promising therapeutic applications beyond the combination’s established use in type 2 diabetes with dyslipidemia. Notably, the top-ranked predicted indications include breast carcinoma and myocardial infarction, suggesting that this drug pairing may have benefits spanning oncologic and cardiovascular domains. Preclinical studies have reported synergistic anti-cancer effects of metformin combined with atorvastatin, particularly in breast cancer, where clinical trials have evaluated the combination’s ability to suppress tumor proliferation, as measured by Ki-67 expression, during the presurgical window between diagnostic biopsy and tumor resection [33]. In cardiovascular settings, randomized controlled trials have demonstrated that the combination improves both glycemic control (HbA1c) and lipid profiles (LDL-cholesterol) more effectively than either agent alone in patients with type 2 diabetes and dyslipidemia [32]. Complementary evidence from diabetic animal models further indicates that combined metformin and atorvastatin therapy attenuates diabetic cardiomyopathy and reduces the risk of myocardial infarction and stroke through coordinated reductions in inflammation, oxidative stress, and apoptosis [34, 35]. These biologically and clinically supported indications were ranked highly by DualKG-DC, suggesting that the framework is able to recover therapeutically relevant and mechanistically plausible drug-combination indications.

**Table 2:** Top 5 ranked potential disease indications predicted for the metformin-atorvastatin combination

No.	Predicted Indication	Mechanistic Rationale	Supporting Evidence
1	Breast Cancer	Targeting cancer cell metabolism, promoting apoptosis, reducing proliferation, and suppressing angiogenesis	NCT01980823
2	Hypertensive diseases		
3	Diabetes Mellitus	Upregulating the AMP-activated protein kinase (AMPK) and Sirtuin 1 (SIRT1) pathways, which are critical for enhancing insulin sensitivity.	NCT02947620 PMID: 20,034,685
4	Myocardial Infarction	Reducing inflammation, oxidative stress, and cardiomyocyte apoptosis	PMID: 33,718,370
5	Stroke disorders		

NCT identifiers were obtained from ClinicalTrials.gov, and PMID were retrieved from PubMed

## Discussion

In this study, we developed DualKG-DC, a computational framework for drug combination prediction. DualKG-DC leveraged a dual-layer knowledge graph and a heterogeneous GNN, which initially learned broad biological information and subsequently refined this knowledge using drug combination-specific data to predict indications for drug combinations. Extensive experiments demonstrated that DualKG-DC outperformed baseline methods, highlighting two major advantages. First, the framework employed a two-stage training strategy involving pretraining on the foundational BiologicalKG and fine-tuning on the task-specific drug combination centric subgraph, which improved prediction accuracy while reducing reliance on large, labeled datasets. Second, the dual-layer architecture enhanced the model’s generalizability, enabling robust performance in cold-start settings involving previously unseen drug combinations.

DualKG-DC has several limitations. First, constructing gold-standard negative samples is challenging due to the lack of known negative associations between drug combinations and diseases in public databases and literature, potentially leading to the misclassification of unknown positive associations as negative. While some negative samples are present in the literature [36, 37] and therefore included in our framework, the underreporting of negative results also limits available data [38]. Second, our current framework focuses on integrating structural data and does not yet include sequence data, such as protein sequences, single cell sequencing data, which could offer valuable biological insights. In future iterations, we aim to improve its predictive capabilities by incorporating models like the Transformer model, specifically designed to learn and extract meaningful patterns from sequence data. Third, the disease ranked by our model require further mechanistic investigation and clinical evaluation to assess their biological plausibility and therapeutic relevance. Lastly, the computational cost and scalability of training and inference on large-scale knowledge graphs and expanding drug combination spaces may pose challenges when extending the framework to larger settings. To address this, we plan to optimize our framework by employing more efficient algorithms, leveraging parallel computing or swarm algorithms [39] to reduce processing time.

For future work, we plan to enhance the interpretability of the DualKG-DC model. Specifically, we plan to focus on extracting and interpreting relational paths within the knowledge graph, leveraging biological prior knowledge to guide this process. By integrating domain-specific knowledge, we hope to provide more transparent and biologically meaningful explanations for the model’s predictions, enabling better understanding and trust in its outcomes. Furthermore, these methods could be expanded to generate time-aware embeddings of drug combinations, following the work of Soman et al. [40]. Such methods could be used to predict long-term efficacy and side effects of drug combinations, reducing type II errors in clinical trials due to insufficient patient follow-up. Incorporating temporal information could also allow for the inclusion of time-series gene expression data and clinical trial timelines to allow the model to capture evolving biological processes and predict long-term treatment outcomes.

In conclusion, DualKG-DC offers a flexible framework for drug combination prediction by leveraging a dual-layer knowledge graph architecture. Its excellent performance in both transductive and inductive settings highlights its effectiveness in capturing complex biomedical relationships and generalizing to novel combinations. Beyond the drug combination task, the DualKG-DC approach demonstrates the broader potential of dual-layer knowledge graph models for addressing other challenges in biomedical research.

## Supplementary Information

Below is the link to the electronic supplementary material.

Supplementary Material 1

## Data Availability

No datasets were generated or analysed during the current study.

## Code Availability

The code and datasets are available at https://github.com/zhenxianggao/DualKG-DC
