# Supplementary material for: DualKG-DC: A Drug-Centric Dual-Layer Knowledge Graph Framework for Drug Combination Prediction
Source: J Med Syst. 2026 May 28;50(1):88. doi: 10.1007/s10916-026-02416-y (PMC13219182; doi:10.1007/s10916-026-02416-y)
Supplement: Supplementary file 1 — Supplementary Material 1 [file 10916_2026_2416_MOESM1_ESM.pdf]

Supporting Information

Table S1. Statistics of Interactions in BiologicalKG

Data Source	Interaction Type	Interaction Number	Node Type	Node Number
DisGeNET	Disease - Gene	99,321	Disease	11,902
			Gene	10,003
Human Phenotype Ontology (HPO)	Disease - HPO	237,830	Disease	10,532
			Human Phenotype Ontology	10,640
	Gene - HPO	238,235	Gene	4,918
			Human Phenotype Ontology	9,600
Mouse Genome Informatics	Gene - Mammalian Phenotype (MP)	79,414	Gene	13,365
			Mammalian Phenotype (MP)	27
DrugBank	Drug - Gene	10,518	Drug	1,928
			Gene	2,423
CTD : Comparative Toxicogenomics Database	Disease - Pathway	176,869	Disease	5,535
			Pathway	317
	Gene - Pathway	19,113	Gene	4,186
			Pathway	317
STRING: functional protein association networks	Gene - Gene	74,394	Gene	10,844

Table S2. Statistics of Text-mined knowledge

Data Source	Interaction Type	Interaction Number	Node Type	Node Number
Text-mined knowledge	Drug - Disease	33,895	Drug	1,680
			Disease	5,208
Text-mined knowledge	Disease – Disease (Comorbidity)	88,783	Disease	12,582
Text-mined knowledge	Drug Combination - Disease	4,933	Drug Combination	2,009
			Disease	1,196
